# Commentary on “Questioning the HIV–AIDS Hypothesis: 30 Years of Dissent”

**DOI:** 10.3389/fpubh.2015.00030

**Published:** 2015-02-13

**Authors:** Seth C. Kalichman

**Affiliations:** ^1^Department of Psychology, University of Connecticut, Storrs, CT, USA

**Keywords:** AIDS denialism, HIV/AIDS, Duesberg, pseudoscience

The Earth is spherical. The holocaust occurred. Man did walk on the moon. Carbon dioxide emissions are warming the planet. And yes, HIV causes AIDS. These truths are established by an accumulation of accepted and irrefutable facts. Nevertheless, individuals are free to deny facts and believe whatever they choose. But believing does not make it so. There are plenty of forums to express erroneous opinions, especially on the Internet (1, 2). AIDS denialists, those who state that HIV is harmless and not the cause of AIDS, have created an abundance of such outlets and are free to spew as much misinformation about this deadly disease as they wish. Occasionally, the filter of scientific peer review has failed and AIDS denialism has made its way into reputable scientific journals. When a journal’s unfortunate error of disseminating AIDS denialism is discovered, publishers and editors have typically taken corrective action through retractions, statements of concern, errata, and explanatory editorials (3–6). I was therefore invited by the publishers of *Frontiers in Public Health* to contribute this comment in response to Patricia Goodson’s article *Questioning the HIV*–*AIDS hypothesis: 30 years of dissent* (7).

At the expense of her own credibility as well as the reputation of *Frontiers in Public Health*, Patricia Goodson has actually performed a public service. It is important for people to know that AIDS denialists do indeed still exist. AIDS denialists are the best known for having joined with former President of South Africa Thabo Mbeki to create the impression that there is a debate among scientists as to whether HIV causes AIDS. Tragically, more than 330,000 South Africans needlessly died and 35,000 babies were born with HIV infection as a result of Mbeki’s denialist policies (8–11). The harms of AIDS denialism continue today and extend beyond the boarders of South Africa. One group that tracks the activities of AIDS denialists, www.AIDSTruth.org post the tragic stories of over 25 prominent AIDS denialists who have died of AIDS. There are numerous testimonials from family members and friends of people who have refused to accept their HIV diagnoses and decline treatment because they have been persuaded by AIDS denialists into thinking their HIV test is invalid, HIV is harmless, and antiretroviral medications are toxic poisons (see www.denyingaids.blogspot.com). For those most vulnerable to medical misinformation, especially people dealing with the trauma of a life threatening medical diagnosis, it can be difficult to distinguish the fraudulent claims of AIDS denialists from credible science and medicine.

Goodson’s article is a primer on AIDS denialism unlike any seen in what is purportedly a peer-reviewed journal. Goodson relies on material found in articles more than two decades old, a time when HIV first emerged and there were legitimate questions raised about a then unknown pathogen. Goodson’s article relies on self-published books, blog posts, essays, and fringe articles. There is no credible research offered by Goodson to support her opinion that there is any debate about HIV as the cause of AIDS, simply because there is no such debate.

Goodson is dismissive of science and medicine. Like the AIDS denialists she gives credence, her views are myopic. Consider her summary of the leading “scientists” who have, for decades, questioned HIV as the cause of AIDS. Sadly, Goodson has not been forthright in her characterization of these characters despite having full awareness of their complete records. She cites my book *Denying AIDS* (12), which discusses both their credentials and credibility. In *Denying AIDS*, I note that at the start of the AIDS crisis there were numerous potential explanations offered to explain this new disease. However, the fact that AIDS is caused by an infectious agent, to become called HIV, was determined within just a few years. But despite the facts, a small group of rogue scientists, headed by Peter Duesberg, held fast to failed theories. Although they consider themselves dissident scientists, they are actually denialists because they utilize specific tactics to evade and deny reality. AIDS denialists were obscure and mostly ignored until the advent of the world-wide-web. The rise of the Internet afforded denialists to amplify their volume and expand their reach. AIDS denialists have created a pseudoscience that has had its own “journals” and has even conducted unethical and illegal human research. While some AIDS denialists have advanced degrees, none has credibility in the scientific community. At best, Goodson has confused AIDS denialists’ credentials with credibility. At worst, she intentionally ignores their notoriety.

For example, Goodson describes Peter Duesberg as a Professor of Molecular and Cell Biology at University of California, Berkeley and a member of the National Academy of Sciences. While it is true that he was part of the group that isolated the first cancer gene and mapped the genetic structure of retroviruses in 1970, Duesberg later claimed, and still claims, that no such genes exist (13). Duesberg has never conducted any research on HIV and he was a leading voice on Mbeki’s infamous South African Presidential AIDS Panel. Goodson describes Duesberg’s close companion David Rasnick as a trained biochemist who worked on protease inhibitors. However, his work was only with rodents and never had anything to do with HIV. Rasnick is a former President of the AIDS denialist group Rethinking AIDS. Rasnick is reported to have convinced Mbeki that there is a scientific debate on the cause of AIDS and suggested that Mbeki outlaw HIV testing and ban antiretroviral drugs (14, 15). Rasnick collaborated with German vitamin salesman Matthias Rath to conduct illegal clinical trials of vitamins as a cure for AIDS. The South African courts have found Rasnick guilty of conducting unlawful clinical trials that may have killed people. Conveniently, Goodson makes no mention of these facts.

It is also true that Kary Mullis was a 1993 Nobel Laureate who developed polymerase chain reaction. What Goodson chooses to ignore is that Mullis is also a self-proclaimed avid user of LSD and tells of how he was abducted by aliens (16). John F. Martin, President of the European Society for Clinical Investigation summed up Mullis by stating “Mullis not only decreased the nobility of the prize but also his attitude was, I believe, a potential corrupting influence on young scientists: among other things, for example, he claimed himself to have changed data-points so as to make data-sets appear more significant by way of illustrating that the practice is a common one.” And finally, Henry Bauer is a Professor and Dean Emeritus at Virginia Tech. He was also Editor-in-Chief of a major outlet for pseudoscience, *Journal of Scientific Exploration* and is a leading authority on the existence of the Loch Ness Monster (17, 18). Goodson relies on other discredited scientists, particularly former President of Rethinking AIDS and member of Mbeki’s AIDS Panel Etienne de Harven, and non-scientists including former math instructor Rebecca Culshaw.

As academics and scientists, our credentials are based on the degrees we receive and the appointments we hold, neither of which are typically revocable. However, our credibility is earned by the sum of our accomplishments, not just those things that we say and write that suit an opinion. When his life’s work is considered in total, are we to believe that Henry Bauer is a serious scientist? Should we conclude that his views on HIV causing AIDS are as credible as his research on the monsters of Loch Ness? Does Dr. Mullis’ Nobel Prize offer him a lifetime pass on all of his views, including those on the use of LSD, faking data, alien abductions, and HIV not causing AIDS? Does being elected to the National Academy of Sciences mean that Peter Duesberg is to be taken seriously when he claims that there are no genetic influences involved in any form of cancer and that HIV is harmless? The full record of a scientist should be taken into account when considering credibility. Publishing an article that blindly gives credence to AIDS denialism is now part of the record for Patricia Goodson and Editor Sanjay Zodpey, at the unfortunate expense of *Frontiers in Public Health*.

## Conflict of Interest Statement

The author declares that the research was conducted in the absence of any commercial or financial relationships that could be construed as a potential conflict of interest.
